# New potent steroid sulphatase inhibitors based on 6-(1-phenyl-1*H*-1,2,3-triazol-4-yl)naphthalen-2-yl sulphamate derivatives

**DOI:** 10.1080/14756366.2020.1858820

**Published:** 2020-12-15

**Authors:** Olga Ciupak, Mateusz Daśko, Karol Biernacki, Janusz Rachon, Maciej Masłyk, Konrad Kubiński, Aleksandra Martyna, Sebastian Demkowicz

**Affiliations:** a Department of Organic Chemistry, Faculty of Chemistry, Gdańsk University of Technology, Gdańsk, Poland; b Department of Inorganic Chemistry, Faculty of Chemistry, Gdańsk University of Technology, Gdańsk, Poland; c Department of Molecular Biology, The John Paul II Catholic University of Lublin, Lublin, Poland

**Keywords:** Steroid sulphatase, hormone-dependent cancer, breast cancer, STS inhibitors, triazoles

## Abstract

In the present work, we report a new class of potent steroid sulphatase (STS) inhibitors based on 6-(1-phenyl-1*H*-1,2,3-triazol-4-yl)naphthalen-2-yl sulphamate derivatives. Within the set of new STS inhibitors, 6-(1-(1,2,3-trifluorophenyl)-1*H*-1,2,3-triazol-4-yl)naphthalen-2-yl sulphamate **3L** demonstrated the highest activity in the enzymatic assay inhibiting the STS activity to 7.98% at 0.5 µM concentration. Furthermore, to verify whether the obtained STS inhibitors are able to pass through the cellular membrane effectively, cell line experiments have been carried out. We found that the lowest STS activities were measured in the presence of compound **3L** (remaining STS activity of 5.22%, 27.48% and 99.0% at 100, 10 and 1 nM concentrations, respectively). The measured STS activities for *Irosustat* (used as a reference) were 5.72%, 12.93% and 16.83% in the same concentration range. Moreover, a determined IC_50_ value of 15.97 nM for **3L** showed that this compound is a very promising candidate for further preclinical investigations.

## Introduction

Cancer is the second leading cause of death worldwide. As reported by *International Agency for Research on Cancer*, there were more than 18 million new cases diagnosed and 9.5 million tumour-related deaths in 2018 worldwide^1^. The most common types of cancer are lung, breast, colorectal, prostate, and stomach, which constitute almost 50% of all tumour cases. Moreover, lung, colorectal, stomach, liver, and breast cancers are responsible for nearly 50% of all deaths. *National Cancer Institute* estimates that more than 270 000 (15.3%) new cases of breast cancer will be diagnosed and more than 42 000 (7.0%) deaths will be reported of this disease in the United States of America in 2020^2^. Approximately 95% of breast cancer cases in the primary stage are hormone-sensitive, and therefore, biological active hormones (including oestrogens and androgens) play a crucial role in the proliferation of tumours cells^3^. The biosynthesis of active steroids in cancer tissues mainly depends on the following three enzymatic pathways: aromatase (responsible for the transformation of androgens into oestrogens), 17β-hydroxysteroid dehydrogenase (implicated in the reduction of oestrone to oestradiol) and steroid sulphatase (STS). STS acts by hydrolysing steroid sulphates (including oestrone sulphate and dehydroepiandrosterone sulphate) and therefore plays a pivotal role in human steroidogenesis process^4–7^. Current breast cancer therapies are based on chemotherapeutics that act as selective oestrogen receptor modulators (SERMs) (e.g. *Tamoxifen*) or inhibitors of the aromatase enzyme complex (e.g. *Letrozole* or *Anastrozole*)^8–10^. Unfortunately, both methods of treatment are unsatisfactory. The main reason for the failure of the therapy based on aromatase inhibitors is the fact that aromatase expression is significantly lower than STS^11^. Furthermore, there are studies demonstrating a significant increment of STS and 17β-HSD1 following aromatase inhibitor therapy of ER positive postmenopausal breast carcinoma patients due to the compensatory response of breast carcinoma tissues to oestrogen depletion^12^.

Nowadays, STS has been considered as an attractive molecular target for the development of hormone-dependent cancer therapies, and therefore, the synthesis of new, efficient, selective STS inhibitors is of particular importance for modern medicinal chemistry. Recently, there has been intensive research towards finding novel STS inhibitors. Scientists have developed, both, steroidal and non-steroidal compounds containing various functional groups (e.g. sulphamate and phosphorus moieties)^13^. For example, one of the most promising drug candidate based on a sulphamoylated coumarin core is *Irosustat* (also known as 667-COUMATE or STX64). *Irosustat* is in clinical trials (phase II clinical studies) and exhibits quite good results towards treating hormone-dependent breast cancer (without having *in vivo* and *in vitro* oestrogenic properties)^14–20^. Although *Irosustat* showed very promising clinical effects in the treatment of hormone-dependent tumours, in relation to endometrium (where occurs high STS activity) *Irosustat* did not demonstrate activity sufficient for future commercial development^21^. For this reason, the search for more effective STS inhibitors is still ongoing.

In the present work, we described our recent research on the design, synthesis, and biological evaluation of compounds based on non-steroidal core containing triazole and naphthalene rings in their structure as new and potent STS inhibitors. The presence of the triazole ring in the structure of obtained compounds is justified by the fact that triazoles are a class of compounds showing very attractive properties and are successfully used in the design of new pharmaceuticals^22^. One of the most important triazoles feature is that they do not undergo hydrolysis under acidic, basic, and redox conditions and they are stable to metabolic degradation. Moreover, these compounds are weakly acidic and weakly basic. Some properties like hydrogen bond formation, π-π stacking interaction, strong dipole moments, and bioisosteric effects make triazole derivatives an attractive type of compounds in the field of medicinal chemistry. Despite the fact that 1,2,3-triazoles do not exist in the nature^23^ such compounds exhibit many biological activities (e.g. anticancer^24^^,^^25^, antimicrobial^26^^,^^27^, anti-infective^28^).

## Materials and methods

### General methods and materials

6-Bromo-2-naphthol, trimethylsilylacetylene, palladium(II) chloride, triphenylphosphine, copper(I) iodide, triethylamine, the appropriate aniline derivatives, *tert*-butyl nitrite (*t*-BuONO), azidotrimethylsilane (TMSN_3_), 1 M solution of tetrabutylammonium fluoride (TBAF) in tetrahydrofuran (THF), sodium ascorbate, copper(II) sulphate pentahydrate, chlorosulphonyl isocyanate, *N*,*N*-dimethylacetamide (*N*,*N*-DMA), and formic acid were commercially available from Sigma-Aldrich. Radiolabelled [^3^H] oestrone sulphate for enzyme and cellular assays was purchased from PerkinElmer. Acetonitrile (ACN) and dichloromethane (DCM) were dried and distilled using standard procedures. Melting points (uncorrected) were determined with a Stuart Scientific SMP30 apparatus. Infra-red spectra were measured on a Nicolet 8700 spectrometer. ^1^H and ^13^C NMR spectra were recorded on a Bruker Avance III HD 400 MHz spectrometer. Chemical shifts δ are reported in parts per million relative to the residual solvent peak (CDCl_3_ = 7.26 ppm for ^1^H, 77.0 ppm for ^13^C, DMSO-d_6_ 2.49 ppm for ^1^H, and 39.5 ppm for ^13^C). Coupling constants are given in Hertz. Mass spectra were recorded on an Agilent 6540 Accurate Mass Q-TOF LC/MS System. Elemental analysis was performed using CHNS-Carlo Erba EA-1108. Preparative thin-layer chromatography (TLC) was carried out with Polygram SIL G/UV254, silica gel (Macherey-Nagel GmbH & Co. KG, Düren, Germany). Column chromatography was performed using silica gel 60 (230-400 mesh, Merck).

#### General method for the synthesis of 6-((trimethylsilyl)ethynyl)naphthalen-2-ol 1

A solution of 6-bromo-2-naphthol (892 mg, 4 mmol), trimethylsilylacetylene (0.831 ml, 6 mmol), palladium(II) chloride (35.5 mg, 0.20 mmol), triphenylphosphine (105 mg, 0.40 mmol), copper(I) iodide (19 mg, 0.10 mmol) and triethylamine (3.94 ml, 28.2 mmol) in dry ACN (20 ml) was refluxed under an argon atmosphere. After 3 h of heating, the reaction mixture was cooled down and filtered. The filtrate was concentrated under reduced pressure and crude product was purified with column chromatography using AcOEt/hexane (1:4) as eluent to afford the desired compound **1**.

**6-((Trimethylsilyl)ethynyl)naphthalen-2-ol 1** Yield 68%; mp 92-94 °C; ν_max_ (KBr)/cm^−1^ 3245, 2166, 1602, 1506, 1248, 941, 836, 703; ^1^H NMR δ_H_ (400 MHz, CDCl_3_) 7.96 (1H, s, Ar-H), 7.73-7.70 (1H, m, Ar-H), 7.61 (1H, d, *J* = 8.5 Hz, Ar-H), 7.49 (1H, dd, *J* = 8.5, 1.6 Hz, Ar-H), 7.14-7.11 (2H, m, Ar-H), 5.55-4.53 (1H, brs, OH) 0.31 (9H, s, CH_3_); ^13^C NMR δ_C_ (101 MHz, CDCl_3_) 154.1, 134.2, 132.0, 129.9, 129.3, 128.3, 126.3, 118.4, 118.1, 109.6, 105.7, 93.8, 0.1; Anal. calcd for: C_15_H_16_OSi, C, 74.95; H, 6.71; O, 6.66; Si, 11.68. Found: C, 74.90; H, 6.75; O, 6.70; Si, 11.68%. HRMS (m/z) [M—H]^–^ calcd 239.1155, found 239.0897.

#### General method for the synthesis of 6–(1-phenyl-1H-1,2,3-triazol-4-yl)naphthalen-2-ol derivatives 2 A-L

To an ice-cooled solution of corresponding amine (2.63 mmol) in ACN (6.1 ml), *t*-BuONO (325 mg, 3.16 mmol) was added dropwise, followed by TMSN_3_ (333 mg, 2.89 mmol). Solution was stirred at room temperature for 4 h and in the next step, **1** (632 mg, 2.63 mmol) and 1 M solution of TBAF in THF (2.89 ml) were added. The reaction mixture was stirred at 0 °C for 30 min. Subsequently, CuSO_4_ · 5H_2_O (65.7 mg, 0.263 mmol) and a freshly prepared aqueous solution (0.526 ml) of sodium ascorbate (104 mg, 0.526 mmol) were added, and the obtained solution was stirred for 24 h under an argon atmosphere at room temperature. Afterwards, the reaction mixture was concentrated under vacuum and the crude product was dissolved in AcOEt (30 ml). Obtained solution was washed with 0.1 M hydrochloric acid. After separation organic phase was dried using MgSO_4_, solvent was evaporated and the resulting residue was crystallised from ACN to give the desired products **2 A-L**.

**6-(1-Phenyl-1*H*-1,2,3-triazol-4-yl)naphthalen-2-ol 2A** Yield 64%; mp (with decomposition) 228-230 °C; ν_max_ (KBr)/cm^−1^ 3293, 3137, 1598, 1504, 1228, 1036, 871, 683; ^1^H NMR δ_H_ (400 MHz, DMSO-d_6_) 9.87 (1H, s, OH), 9.35 (1H, s, CH), 8.39 (1H, s, Ar-H), 8.02-7.96 (3H, m, Ar-H), 7.86 (1H, d, *J* = 8.8 Hz, Ar-H), 7.82 (1H, d, *J* = 8.7 Hz, Ar-H), 7.65 (2H, t, *J* = 7.9 Hz, Ar-H), 7.53 (1H, t, *J* = 7.4 Hz, Ar-H), 7.18 (1H, d, *J* = 2.3 Hz, Ar-H), 7.15 (1H, dd, *J* = 8.7, 2.4 Hz, Ar-H); ^13^C NMR δ_C_ (101 MHz, DMSO-d_6_) 156.2, 148.2, 137.2, 134.9, 130.4, 130.2, 129.1, 128.2, 127.3, 125.0, 124.4, 124.3, 120.4, 119.8, 119.7, 109.3; Anal. calcd for: C_18_H_13_N_3_O, C, 75.25; H, 4.56; N, 14.63; O, 5.57. Found: C, 75.31; H, 4.49; N, 14.71; O, 5.49%. HRMS (m/z) [M + H]^+^ calcd 288.1132, found 288.3504.

**6-(1-(3-Fluorophenyl)-1*H*-1,2,3-triazol-4-yl)naphthalen-2-ol 2B** Yield 43%; mp (with decomposition) 219-220 °C; ν_max_ (KBr)/cm^−1^ 3251, 3125, 2352, 1600, 1498, 1231, 1033, 870, 677; ^1^H NMR δ_H_ (400 MHz, DMSO-d_6_) 9.88 (1H, s, OH), 9.40 (1H, s, CH), 8.37 (1H, s, Ar-H), 7.95 (1H, dd, *J* = 8.5, 1.7 Hz, Ar-H), 7.93-7.80 (4H, m, Ar-H), 7.70 (1H, td, *J* = 8.3, 6.5 Hz, Ar-H), 7.38 (1H, tdd, *J* = 8.5, 2.5, 0.7 Hz, Ar-H), 7.18 (1H, d, *J* = 2.1 Hz, Ar-H), 7.15 (1H, dd, *J* = 8.7, 2.4 Hz, Ar-H); ^13^C NMR δ_C_ (101 MHz, DMSO-d_6_) 162.9 (d, ^1^*J*_C-F_ = 245.1 Hz), 156.3, 148.3, 138.4 (d, ^3^*J*_C-F_ = 10.5 Hz), 134.9, 132.4 (d, ^3^*J*_C-F_ = 9.2 Hz), 130.2, 128.1, 127.3, 124.8, 124.3, 119.9, 119.8, 116.3 (d, ^4^*J*_C-F_ = 3.0 Hz), 115.8 (d, ^2^*J*_C-F_ = 21.0 Hz), 109.3, 107.9 (d, ^2^*J*_C-F_ = 26.5 Hz); Anal. calcd for: C_18_H_12_FN_3_O, C, 70.81; H, 3.96; F, 6.22; N, 13.76; O, 5.24. Found: C, 70.79; H, 3.99; F, 6.24; N, 13.69; O, 5.29%. HRMS (m/z) [M + H]^+^ calcd 306.1038, found 306.3540.

**6-(1-(3-Chlorophenyl)-1*H*-1,2,3-triazol-4-yl)naphthalen-2-ol 2C** Yield 45%; mp (with decomposition) 229-231 °C; ν_max_ (KBr)/cm^−1^ 3254, 3129, 2345, 1598, 1221, 1035, 869, 679; ^1^H NMR δ_H_ (400 MHz, DMSO-d_6_) 9.88 (1H, s, OH), 9.43 (1H, s, CH), 8.37 (1H, s, Ar-H), 8.12 (1H, t, *J* = 2.0 Hz, Ar-H), 8.01 (1H, ddd, *J* = 8.1, 2.0, 0.9 Hz, Ar-H), 7.95 (1H, dd, *J* = 8.5, 1.7 Hz, Ar-H), 7.86 (1H, d, *J* = 8.8 Hz, Ar-H), 7.82 (1H, d, *J* = 8.6 Hz, Ar-H) 7.68 (1H, t, *J* = 8.1 Hz, Ar-H), 7.59 (1H, ddd, *J* = 8.1, 1.9, 0.9 Hz, Ar-H), 7.19-7.17 (1H, m, Ar-H), 7.15 (1H, dd, *J* = 8.7, 2.4 Hz, Ar-H); ^13^C NMR δ_C_ (101 MHz, DMSO-d_6_) 156.3, 148.3, 138.2, 134.9, 134.7, 132.2, 130.2, 128.9, 128.1, 127.3, 124.8, 124.3, 120.2, 119.9, 119.8, 118.9, 109.3; Anal. calcd for: C_18_H_12_ClN_3_O, C, 67.19; H, 3.76; Cl, 11.02; N, 13.06; O, 4.97. Found: C, 67.09; H, 3.84; Cl, 11.09; N, 12.99; O, 4.99%. HRMS (m/z) [M + H]^+^ calcd 321.0669, found 322.3397.

**6-(1-(3-Bromophenyl)-1*H*-1,2,3-triazol-4-yl)naphthalen-2-ol 2D** Yield 42%; mp (with decomposition) 226-228 °C; ν_max_ (KBr)/cm^−1^ 3271, 3130, 2922, 2350, 1583, 1489, 1034, 782; ^1^H NMR δ_H_ (400 MHz, DMSO-d_6_) 9.88 (1H, s, OH), 9.43 (1H, s, CH), 8.37 (1H, s, Ar-H), 8.24 (1H, s, Ar-H), 8.04 (1H, d, *J* = 8.1 Hz, Ar-H), 7.95 (1H, d, *J* = 8.2 Hz, Ar-H), 7.86 (1H, d, *J* = 8.8 Hz, Ar-H), 7.82 (1H, d, *J* = 8.6 Hz, Ar-H), 7.72 (1H, d, *J* = 7.8 Hz, Ar-H), 7.61 (1H, t, *J* = 8.0 Hz, Ar-H), 7.18 (1H, s, Ar-H), 7.15 (1H, d, *J* = 8.9 Hz, Ar-H); ^13^C NMR δ_C_ (101 MHz, DMSO-d_6_) 156.3, 148.3, 138.3, 134.9, 132.4, 131.8, 130.2, 128.1, 127.3, 124.8, 124.3, 123.0, 122.9, 119.9, 119.8, 119.3, 109.3; Anal. calcd for: C_18_H_12_BrN_3_O, C, 59.03; H, 3.30; Br, 21.82; N, 11.47; O, 4.37. Found: C, 59.11; H, 3.28; Br, 21.87; N, 11.50; O, 4.24%. HRMS (m/z) [M + H]^+^ calcd 366.0237, found 366.3458.

**6-(1-(4-Fluorophenyl)-1*H*-1,2,3-triazol-4-yl)naphthalen-2-ol 2E** Yield 74%; mp (with decomposition) 237-239 °C; ν_max_ (KBr)/cm^−1^ 3651, 3199, 3124, 2920, 2347, 1614, 1507, 1039, 802; ^1^H NMR δ_H_ (400 MHz, DMSO-d_6_) 9.86 (1H, s, OH), 9.33 (1H, s, CH), 8.37 (1H, s, Ar-H), 8.06-8.00 (2H, m, Ar-H), 7.96 (1H, dd, *J* = 8.5, 1.5 Hz, Ar-H), 7.86 (1H, d, *J* = 8.8 Hz, Ar-H), 7.82 (1H, d, *J* = 8.6 Hz, Ar-H), 7.52 (2H, t, *J* = 8.8 Hz, Ar-H), 7.17 (1H, s, Ar-H), 7.15 (1H, dd, *J* = 8.8, 2.3 Hz, Ar-H); ^13^C NMR δ_C_ (101 MHz, DMSO-d_6_) 162.1 (d, ^1^J_C-F_ = 245.8 Hz), 156.2, 148.2, 134.9, 133.8 (d, ^4^J_C-F_ = 2.8 Hz), 130.1, 128.2, 127.3, 125.0, 124.4, 124.2, 122.8 (d, ^3^J_C-F_ = 8.8 Hz), 120.0, 119.8, 117.3 (d, ^2^J_C-F_ = 23.3 Hz), 109.2; Anal. calcd for: C_18_H_12_FN_3_O, C, 70.81; H, 3.96; F, 6.22; N, 13.76; O, 5.24. Found: C, 70.75; H, 3.99; F, 6.27; N, 13.81; O, 5.18%. HRMS (m/z) [M + H]^+^ calcd 306.1038, found 306.3524.

**6-(1-(4-Chlorophenyl)-1*H*-1,2,3-triazol-4-yl)naphthalen-2-ol 2F** Yield 74%; mp (with decomposition) 256-257 °C; ν_max_ (KBr)/cm^−1^ 3286, 3119, 2294, 1611, 1550, 1032, 831; ^1^H NMR δ_H_ (400 MHz, DMSO-d_6_) 9.86 (1H, s, OH), 9.38 (1H, s, CH), 8.37 (1H, s, Ar-H) 8.03 (2H, d, *J* = 8.9 Hz, Ar-H), 7.95 (1H, dd, *J* = 8.5, 1.6 Hz, Ar-H), 7.86 (1H, d, *J* = 8.8 Hz, Ar-H), 7.82 (1H, d, *J* = 8.6 Hz, Ar-H), 7.73 (2H, d, *J* = 8.9 Hz, Ar-H), 7.17 (1H, s, Ar-H), 7.15 (1H, dd, *J* = 8.7, 2.3 Hz, Ar-H); ^13^C NMR δ_C_ (101 MHz, DMSO-d_6_) 156.3, 148.3, 136.0, 134.9, 133.4, 130.4, 130.2, 128.2, 127.3, 124.9, 124.4, 124.3, 122.1, 119.8, 109.3; Anal. calcd for: C_18_H_12_ClN_3_O, C, 67.19; H, 3.76; Cl, 11.02; N, 13.06; O, 4.97. Found: C, 67.24; H, 3.69; Cl, 11.09; N, 13.04; O, 4.49%. HRMS (m/z) [M + H]^+^ calcd 322.0742, found 322.3345.

**6-(1-(4-Bromophenyl)-1*H*-1,2,3-triazol-4-yl)naphthalen-2-ol 2G** Yield 62%; mp (with decomposition) 254-256 °C; ν_max_ (KBr)/cm^−1^ 3470, 3119, 1612, 1495, 1200, 1030, 867, 810; ^1^H NMR δ_H_ (400 MHz, DMSO-d_6_) 9.86 (1H, s, OH), 9.38 (1H, s, CH), 8.37 (1H, s, Ar-H), 7.99-7.93 (3H, m, Ar-H), 7.88-7.80 (4H, m, Ar-H), 7.17 (1H, s, Ar-H), 7.15 (1H, dd, *J* = 8.7, 2.4 Hz, Ar-H); ^13^C NMR δ_C_ (101 MHz, DMSO-d_6_) 156.3, 148.3, 136.4, 134.9, 133.3, 130.2, 128.1, 127.3, 124.9, 124.4, 124.3, 122.3, 121.7, 119.8, 119.7, 109.3; Anal. calcd for: C_18_H_12_BrN_3_O, C, 59.03; H, 3.30; Br, 21.82; N, 11.47; O, 4.37. Found: C, 59.07; H, 3.32; Br, 21.78; N, 11.54; O, 4.29%. HRMS (m/z) [M + H]^+^ calcd 366.0237, found 366.3154.

**6-(1-(2-Fluorophenyl)-1*H*-1,2,3-triazol-4-yl)naphthalen-2-ol 2H** Yield 47%; mp (with decomposition) 207-208 °C; ν_max_ (KBr)/cm^−1^ 3177, 1614, 1511, 1048, 864, 819; ^1^H NMR δ_H_ (400 MHz, DMSO-d_6_) 9.86 (1H, s, OH), 9.12 (1H, d, *J* = 1.9 Hz, CH), 8.41 (1H, s, Ar-H), 8.02-7.91 (2H, m, Ar-H), 7.83 (2H, dd, *J* = 20.5, 8.7 Hz, Ar-H), 7.68-7.58 (2H, m, Ar-H), 7.54-7.45 (1H, m, Ar-H), 7.19-7.09 (2H, m, Ar-H); ^13^C NMR δ_C_ (101 MHz, DMSO-d_6_) 156.3, 154.3 (d, ^1^*J*_C-F_ = 250.8 Hz), 147.8, 134.8, 131.8 (d,

^2^*J*_C-F_ = 7.8 Hz), 130.2, 128.2, 127.3, 126.5, 126.1 (d, ^3^*J*_C-F_ = 3.7 Hz), 125.3 (d, ^2^*J*_C-F_ = 11.0 Hz), 124.8, 124.4, 122.9 (d, ^3^*J*_C-F_ = 4.1 Hz), 119.8, 117.8, 117.6, 108.9; Anal. calcd for: C_18_H_12_FN_3_O, C, 70.81; H, 3.96; F, 6.22; N, 13.76; O, 5.24. Found: C, 70.78; H, 3.99; F, 6.28; N, 13.70; O, 5.25%. HRMS (m/z) [M + H]^+^ calcd 306.1038, found 306.3529.

**6-(1-(2-Chlorophenyl)-1*H*-1,2,3-triazol-4-yl)naphthalen-2-ol 2I** Yield 62%; mp 104-106 °C; ν_max_ (KBr)/cm^−1^ 3133, 1616, 1493, 1493, 1196, 1050, 862, 804; ^1^H NMR δ_H_ (400 MHz, DMSO-d_6_) 9.86 (1H, s, OH), 9.08 (1H, s, CH), 8.39 (1H, s, Ar-H), 7.96 (1H, dd, *J* = 8.5, 1.6 Hz, Ar-H), 7.90-7.75 (4H, m, Ar-H), 7.73–7.59 (2H, m, Ar-H), 7.21-7.11 (2H, m, Ar-H); ^13^C NMR δ_C_ (101 MHz, DMSO-d_6_) 156.3, 147.4, 135.1, 134.9, 132.2, 131.1, 130.2, 129.0, 128.9, 128.8, 128.2, 127.3, 124.9, 124.4, 124.3, 123.7, 119.8, 109.3; Anal. calcd for: C_18_H_12_ClN_3_O, C, 67.19; H, 3.76; Cl, 11.02; N, 13.06; O, 4.97. Found: C, 67.22; H, 3.70; Cl, 11.08; N, 13.11; O, 4.89%. HRMS (m/z) [M + H]^+^ calcd 322.0742, found 322.3377.

**6-(1-(2-Bromophenyl)-1*H*-1,2,3-triazol-4-yl)naphthalen-2-ol 2J** Yield 68%; mp 105-107 °C; ν_max_ (KBr)/cm^−1^ 3135, 1611, 1493, 1283, 1048, 909; ^1^H NMR δ_H_ (400 MHz, DMSO-d_6_) 9.86 (1H, s, OH), 9.05 (1H, s, CH), 8.39 (1H, s, Ar-H), 7.97-7.95 (2H, m, Ar-H), 7.83 (2H, dd, *J* = 18.6, 8.7 Hz, Ar-H), 7.77 (1H, dd, *J* = 7.8, 1.6 Hz), 7.67 (1H, td, *J* = 7.7, 1.4 Hz, Ar-H), 7.60 (1H, td, *J* = 7.7, 1.4 Hz, Ar-H), 7.21-7.18 (1H, m, Ar-H), 7.14 (1H, dd, *J* = 8.8, 2.4 Hz, Ar-H); ^13^C NMR δ_C_ (101 MHz, DMSO-d_6_) 156.2, 147.3, 136.8, 134.9, 134.2, 132.5, 130.2, 129.5, 129.2, 128.2, 127.3, 125.0, 124.4, 124.3, 123.7, 119.8, 119.4, 109.3; Anal. calcd for: C_18_H_12_BrN_3_O, C, 59.03; H, 3.30; Br, 21.82; N, 11.47; O, 4.37. Found: C, 59.10; H, 3.26; Br, 21.86; N, 11.36; O, 4,42%. HRMS (m/z) [M + H]^+^ calcd 366.0237, found 366.3130.

**6-(1-(3,5-Difluorophenyl)-1*H*-1,2,3-triazol-4-yl)naphthalen-2-ol 2K** Yield 39%; mp (with decomposition) 210-212 °C; ν_max_ (KBr)/cm^−1^ 3128, 1607, 1482, 1303, 1050, 911; ^1^H NMR δ_H_ (400 MHz, DMSO-d_6_) 9.88 (1H, s, OH), 9.43 (1H, s, CH), 8.35 (1H, s, Ar-H), 7.95-7.80 (4H, m, Ar-H), 7.46 (1H, tt, *J* = 9.2, 2.2 Hz, Ar-H), 7.20-7.17 (2H, m, Ar-H), 7.15 (1H, dd, *J* = 8.7, 2.4 Hz, Ar-H); ^13^C NMR δ_C_ (101 MHz, DMSO-d_6_) 163.2 (dd, ^1^*J*_C-F_ = 246.9, ^3^*J*_C-F_ = 14.6 Hz), 156.4, 148.4, 138.9 (t, ^3^*J*_C-F_ = 13.3 Hz), 135.0, 130.2, 128.1, 127.4, 124.6, 124.4, 124.2, 120.0, 119.9, 109.3, 104.4, 104; Anal. calcd for: C_18_H_11_F_2_N_3_O, C, 66.87; H, 3.43; F, 11.75; N, 13.00; O, 4.95. Found: C, 66.90; H, 3.39; F, 11.83; N, 12.96; O, 4.92%. HRMS (m/z) [M + H]+ calcd 324.0943, found. 324.3554.

**6-(1-(2,3,4-Trifluorophenyl)-1*H*-1,2,3-triazol-4-yl)naphthalen-2-ol 2L** Yield 13%; mp (with decomposition) 212-214 °C; ν_max_ (KBr)/cm^−1^ 3175, 1630, 1509, 1305, 1043, 912; ^1^H NMR δ_H_ (400 MHz, DMSO-d_6_) 9.88 (1H, s, OH), 9.14 (1H, d, *J* = 1.7 Hz, CH), 8.40 (1H, s, Ar-H), 7.97 (1H, dd, *J* = 8.5, 1.6 Hz, Ar-H), 7.89-7.77 (3H, m, Ar-H), 7.65 (1H, qd, *J* = 8.5, 1.6 Hz, Ar-H), 7.18-7.16 (1H, m, Ar-H), 7.14 (1H, dd, *J* = 8.7, 2.3 Hz, Ar-H); ^13^C NMR δ_C_ (101 MHz, DMSO-d_6_) 156.3, 152.4-152.0 (m), 149.8-149.5 (m), 147.9, 145.8-145.4 (m), 143.2-142.9 (m), 141.6-141.2 (m), 139.2-138.7 (m), 135.0, 130.2, 128.1, 127.3, 124.5, 124.5, 124.4, 123.1 (dd, ^3^*J*_C-F_ = 8.3, ^4^*J*_C-F_ = 3.7 Hz), 122.9 (d, ^4^*J*_C-F_ = 3.3 Hz), 121.0 (dd, ^3^*J*_C-F_ = 8.5, ^4^*J*_C-F_ = 3.9 Hz), 119.8, 113.8 (dd, ^2^*J*_C-F_ = 18.6, ^3^*J*_C-F_ = 3.8 Hz), 109.; Anal. calcd for: C_18_H_10_F_3_N_3_O, C, 63.35; H, 2.95; F, 16.70; N, 12.31; O, 4.69. Found: C, 63.41; H, 2.89; F, 16.78; N, 12.39; O, 4.53%. HRMS (m/z) [M + H]^+^ calcd 342.0849, found. 342.3574.

#### General method for the synthesis of 6-(1-phenyl-1H-1,2,3-triazol-4-yl)naphthalen-2-yl sulphamate derivatives 3A-L

A solution of chlorosulphonyl isocyanate (212.0 mg, 1.50 mmol) in anhydrous DCM (0.5 ml) was prepared. In the next step, a solution of formic acid (70.9 mg, 1.54 mmol) and *N*,*N*-DMA (1.4 mg, 0.016 mmol) was added, and the reaction mixture was stirred at 40 °C for 3.5 h. Then, a solution of the corresponding derivative **2A-L** (1.00 mmol) in *N*,*N*-DMA (3.4 ml) was added, and the obtained solution was stirred at room temperature overnight. Subsequently, reaction mixture was poured into water (50 ml). The resulting precipitate was filtered and washed with water. The crude product was recrystallized from ACN to give the desired products **3A-L**.

**6-(1-Phenyl-1*H*-1,2,3-triazol-4-yl)naphthalen-2-yl sulphamate 3A** Yield 70%; mp (with decomposition) 210-211 °C; νmax (KBr)/cm^−1^ 3119, 2915, 2358, 1595, 1352, 1168, 759, 672; ^1^H NMR δ_H_ (400 MHz, DMSO-d_6_) 9.47 (1H, s, CH), 8.59 (1H, s, Ar-H), 8.16 (1H, dd, *J* = 8.6, 1.5 Hz, Ar-H), 8.15-8.10 (4H, m, Ar-H, NH_2_), 8.00 (2H, d, *J* = 7.6 Hz, Ar-H), 7.88 (1H, d, *J* = 2.2 Hz, Ar-H), 7.67 (2H, t, *J* = 7.9 Hz, Ar-H), 7.54 (1H, t, *J* = 7.4 Hz, Ar-H), 7.51 (1H, dd, *J* = 8.9, 2.4 Hz, Ar-H); ^13^C NMR δ_C_ (101 MHz, DMSO-d_6_) 148.5, 147.6, 137.1, 133.4, 131.9, 130.5, 130.4, 129.3, 129.1, 128.4, 125.0, 124.0, 122.9, 120.6, 120.5, 119.8; Anal. calcd for: C_18_H_14_N_4_O_3_S, C, 59.01; H, 3.85; N, 15.29; O, 13.10; S, 8.75. Found: C, 58.98; H, 3.88; N, 15.34; O, 13.12; S, 8.68%. HRMS (m/z) [M + H]^+^ calcd 367.0860, found 367.3750.

**6-(1-(3-Fluorophenyl)-1*H*-1,2,3-triazol-4-yl)naphthalen-2-yl sulphamate 3B** Yield 43%; mp (with decomposition) 210-212 °C; ν_max_ (KBr)/cm^−1^ 3120, 2560, 1600, 1358, 1173, 782, 673; ^1^H NMR δ_H_ (400 MHz, DMSO-d_6_) 9.52 (1H, s, CH), 8.58 (1H, s, Ar-H), 8.18-8.06 (5H, m, Ar-H, NH_2_), 7.96-7.86 (3H, m, Ar-H), 7.72 (1H, q, *J* = 7.4 Hz, Ar-H), 7.50 (1H, dd, *J* = 8.9, 2.2 Hz, Ar-H), 7.41 (1H, td, *J* = 8.7, 2.3 Hz, Ar-H); ^13^C NMR δ_C_ (101 MHz, DMSO-d_6_) 162.9 (d, ^1^*J*_C-F_ = 245.2 Hz), 148.6, 147.7, 138.3, (d, ^3^*J*_C-F_ = 10.5 Hz), 133.5, 132.4 (d, ^3^*J*_C-F_ = 9.1 Hz), 131.9, 130.5, 129.2, 128.2, 125.0, 124.1, 122.9, 120.8, 119.8, 116.4 (d, ^4^*J*_C-F_ = 3.0 Hz), 116.0 (d, ^2^*J*_C-F_ = 21.0 Hz), 108.0 (d, ^2^*J*_C-F_ = 26.5 Hz); Anal. calcd for: C_18_H_13_FN_4_O_3_S, C, 56.24; H, 3.41; F, 4.94; N, 14.58; O, 12.49; S, 8.34. Found: C, 56.30; H, 3.45; F, 4.90; N, 14.60; O, 12.41; S, 8,43%. HRMS (m/z) [M + H]^+^ calcd 385.0766, found 385.3810.

**6-(1-(3-Chlorophenyl)-1*H*-1,2,3-triazol-4-yl)naphthalen-2-yl sulphamate 3C** Yield 67%; mp (with decomposition) 214-216 °C; ν_max_ (KBr)/cm^−1^ 3132, 2915, 2376, 1591, 1381, 1177, 784, 676; ^1^H NMR δ_H_ (400 MHz, DMSO-d_6_) 9.53 (1H, s, CH), 8.57 (1H, s, Ar-H), 8.16-8.07 (6H, m, Ar-H, NH_2_), 8.01 (1H, dd, *J* = 7.9, 1.2 Hz, Ar-H), 7.88 (1H, d, *J* = 2.1 Hz, Ar-H), 7.69 (1H, t, *J* = 8.1 Hz, Ar-H), 7.61 (1H, dd, *J* = 8.0, 0.8 Hz, Ar-H), 7.51 (1H, dd, *J* = 8.9, 2.3 Hz, Ar-H); ^13^C NMR δ_C_ (101 MHz, DMSO-d_6_) 148.5, 147.7, 138.1, 134.7, 133.5, 132.2, 131.9, 130.5, 129.2, 129.0, 128.2, 124.9, 124.1, 122.9, 120.8, 120.3, 119.8, 119.0; Anal. calcd for: C_18_H_13_ClN_4_O_3_S, C, 53.94; H, 3.27; Cl, 8.84; N, 13.98; O, 11.97; S, 8.00. Found: C, 53.98; H, 3.29; Cl, 8.80; N, 13.94; O, 11.92; S, 8.07%. HRMS (m/z) [M + H]^+^ calcd 401.0470, found 401.3646.

**6-(1-(3-Bromophenyl)-1*H*-1,2,3-triazol-4-yl)naphthalen-2-yl sulphamate 3D** Yield 34%; mp (with decomposition) 219-221 °C; ν_max_ (KBr)/cm^−1^ 3298, 3171, 2363, 1484, 1368, 1182, 800; ^1^H NMR δ_H_ (400 MHz, DMSO-d_6_) 9.55 (1H, s, CH), 8.58 (1H, s, Ar-H), 8.26 (1H, t, *J* = 1.8 Hz, Ar-H), 8.16-8.09 (5H, m, Ar-H, NH_2_), 8.06 (1H, dd, *J* = 8.1, 1.2 Hz, Ar-H), 7.88 (1H, d, *J* = 2.1 Hz, Ar-H), 7.75 (1H, d, *J* = 8.0 Hz, Ar-H), 7.63 (1H, t, *J* = 8.1 Hz, Ar-H), 7.50 (1H, dd, *J* = 8.9, 2.3 Hz, Ar-H); ^13^C NMR δ_C_ (101 MHz, DMSO-d_6_) 148.6, 147.7, 138.2, 133.5, 132.4, 132.0, 131.9, 130.5, 129.2, 128.2, 124.9, 124.1, 123.0, 122.99, 122.90, 120.9, 119.8, 119.4; Anal. calcd for: C_18_H_13_BrN_4_O_3_S, C, 48.55; H, 2.94; Br, 17.94; N, 12.58; O, 10.78; S, 7.20. Found: C, 48.59; H, 2.96; Br, 17.90; N, 12.55; O, 10.74; S, 7.26%. HRMS (m/z) [M + H]^+^ calcd 444.9965, found 445.3408.

**6-(1-(4-Fluorophenyl)-1*H*-1,2,3-triazol-4-yl)naphthalen-2-yl sulphamate 3E** Yield 36%; mp (with decomposition) 226-227 °C; ν_max_ (KBr)/cm^−1^ 3314, 3159, 1515, 1357, 1180, 838; ^1^H NMR δ_H_ (400 MHz, DMSO-d_6_) 9.44 (1H, s, CH), 8.57 (1H, s, Ar-H), 8.17-8.09 (5H, m, Ar-H, NH_2_), 8.07-8.01 (2H, m, Ar-H), 7.88 (1H, dd, *J* = 2.0 Hz, Ar-H), 7.58-7.46 (3H, m, Ar-H); ^13^C NMR δ_C_ (101 MHz, DMSO-d_6_) 162.2 (d, ^1^*J*_C-F_ = 245.9 Hz), 148.5, 147.6, 133.7 (d, ^4^*J*_C-F_ = 2.8 Hz), 133.4, 131.9, 130.5, 129.1, 128.4, 125.0, 124.0, 122.9 (d, ^3^*J*_C-F_ = 5.0 Hz), 122.8, 120.9, 119.8, 117.3 (d, ^2^*J*_C-F_ = 23.3 Hz); Anal. calcd for: C_18_H_13_FN_4_O_3_S, C, 56.24; H, 3.41; F, 4.94; N, 14.58; O, 12.49; S, 8.34. Found: C, 56.27; H, 3.37; F, 4.93; N, 14.62; O, 12.53; S, 8.28%. HRMS (m/z) [M + H]^+^ calcd 385.0766, found 385.3823.

**6-(1-(4-Chlorophenyl)-1*H*-1,2,3-triazol-4-yl)naphthalen-2-yl sulphamate 3F** Yield 40%; mp (with decomposition) 226-229 °C; ν_max_ (KBr)/cm^−1^ 3372, 3269, 1502, 1352, 1173, 813; ^1^H NMR δ_H_ (400 MHz, DMSO-d_6_) 9.49 (1H, s, CH), 8.58 (1H, s, Ar-H), 8.15-8.09 (5H, m, Ar-H, NH_2_), 8.04 (2H, d, *J* = 8.9 Hz, Ar-H), 7.88 (1H, d, *J* = 2.2 Hz, Ar-H), 7.75 (2H, d, *J* = 8.8 Hz, Ar-H), 7.50 (1H, dd, *J* = 8.9, 2.4 Hz, Ar-H); ^13^C NMR δ_C_ (101 MHz, DMSO-d_6_) 148.5, 147.7, 135.9, 133.52, 133.48, 131.9, 130.5, 130.4, 129.2, 128.3, 125.0, 124.1, 122.9, 122.2, 120.7, 119.7; Anal. calcd for: C_18_H_13_ClN_4_O_3_S, C, 53.94; H, 3.27; Cl, 8.84; N, 13.98; O, 11.97; S, 8.00. Found: C, 53.90; H, 3.31; Cl, 8.91; N, 14.01; O, 11.94; S, 7.93%. HRMS (m/z) [M + H]^+^ calcd 401.0470, found 401.3596.

**6-(1-(4-Bromophenyl)-1*H*-1,2,3-triazol-4-yl)naphthalen-2-yl sulphamate 3G** Yield 45%; mp (with decomposition) 234-235 °C; ν_max_ (KBr)/cm^−1^ 3378, 3273, 3143, 1498, 1144, 710; ^1^H NMR δ_H_ (400 MHz, DMSO-d_6_) 9.50 (1H, s, CH), 8.58 (1H, s, Ar-H), 8.16-8.10 (5H, m, Ar-H, NH_2_), 7.97 (2H, d, *J* = 8.9 Hz, Ar-H), 7.89-7.86 (3H, m, Ar-H), 7.50 (1H, dd, *J* = 8.9, 2.3 Hz, Ar-H); ^13^C NMR δ_C_ (101 MHz, DMSO-d_6_) 148.5, 147.7, 136.3, 133.5, 133.4, 131.9, 130.5, 129.2, 128.2, 125.0, 124.1, 122.9, 122.4, 121.9, 120.7, 119.7; Anal. calcd for: C_18_H_13_BrN_4_O_3_S, C, 48.55; H, 2.94; Br, 17.94; N, 12.58; O, 10.78; S, 7.20. Found: C, 48.59; H, 2.90; Br, 17.97; N, 12.61; O, 10.81; S, 7.12%. HRMS (m/z) [M + H]^+^ calcd 445.3490, found 446.9945.

**6-(1-(2-Fluorophenyl)-1*H*-1,2,3-triazol-4-yl)naphthalen-2-yl sulphamate 3H** Yield 34%; mp (with decomposition) 206-207 °C; ν_max_ (KBr)/cm^−1^ 3398, 3277, 3148, 1502, 1111, 728; ^1^H NMR δ_H_ (400 MHz, DMSO-d_6_) 9.50 (1H, d, *J* = 1.8 Hz, CH), 8.61 (1H, s, Ar-H), 8.21-8.06 (5H, m, Ar-H, NH_2_), 7.95 (1H, td, *J* = 7.9, 1.1 Hz, Ar-H), 7.88 (1H, d, *J* = 2.0 Hz, Ar-H), 7.70-7.60 (2H, m, Ar-H), 7.55-7.45 (2H, m, Ar-H); ^13^C NMR δ_C_ (101 MHz, DMSO-d_6_) 154.5 (d, ^1^*J*_C-F_ = 250.9 Hz), 148.5, 147.2, 133.5, 131.9, 130.5, 129.1, 128.2, 126.5, 126.1 (d, ^3^*J*_C-F_ = 3.8 Hz), 125.3 (d, ^2^*J*_C-F_ = 10.5 Hz), 125.1, 124.1, 123.8 (d, ^3^*J*_C-F_ = 4.1 Hz), 122.9, 119.7, 117.8, 117.6; Anal. calcd for: C_18_H_13_FN_4_O_3_S, C, 56.24; H, 3.41; F, 4.94; N, 14.58; O, 12.49; S, 8.34. Found: C, 56.31; H, 3.39; F, 4.90; N,14.56; O, 12.53; S, 8.31%. HRMS (m/z) [M + H]^+^ calcd 385.0766, found 385.3785.

**6-(1-(2-Bromophenyl)-1*H*-1,2,3-triazol-4-yl)naphthalen-2-yl sulphamate 3J** Yield 52%; mp (with decomposition) 188-189 °C; ν_max_ (KBr)/cm^−1^ 3307, 3146, 3052, 1605, 1491, 1370, 929; ^1^H NMR δ_H_ (400 MHz, DMSO-d_6_) 9.19 (1H, s, CH), 8.60 (1H, s, Ar-H), 8.20-8.06 (5H, m, Ar-H, NH_2_), 7.98 (1H, dd, *J* = 8.0, 1.3 Hz, Ar-H), 7.88 (1H, d, *J* = 2.2 Hz, Ar-H), 7.79 (1H, dd, *J* = 7.8, 1.6 Hz, Ar-H), 7.68 (1H, td, *J* = 7.6, 1.4 Hz, Ar-H), 7.61 (1H, td, *J* = 7.7, 1.7 Hz, Ar-H), 7.50 (1H, dd, *J* = 8.9, 2.4 Hz, Ar-H); ^13^C NMR δ_C_ (101 MHz, DMSO-d_6_) 153.7, 150.4, 137.9, 133.1, 132.2, 132.1, 131.4, 129.0, 128.9, 128.5, 126.2, 125.8, 124.7, 122.1, 120.7, 118.7, 115.5, 114.9; Anal. calcd for: C_18_H_13_BrN_4_O_3_S, C, 48.55; H, 2.94; Br, 17.94; N, 12.58; O, 10.78; S, 7.20. Found: C, 48.59, H, 2.96; Br, 17.93; N, 12.57; O, 10.72; S, 7.23%. HRMS (m/z) [M + H]^+^ calcd 444.9965, found. 445.3399

**6-(1-(3,5-Difluorophenyl)-1*H*-1,2,3-triazol-4-yl)naphthalen-2-yl sulphamate 3K** Yield 37%; mp (with decomposition) 214-215 °C; ν_max_ (KBr)/cm^−1^ 3296, 3153, 3052, 1627, 1473, 1357, 916; ^1^H NMR δ_H_ (400 MHz, DMSO-d_6_) 9.55 (1H, s, CH), 8.56 (1H, s, Ar-H), 8.19-8.05 (5H, m, Ar-H, NH_2_), 7.88 (1H, d, *J* = 2.1 Hz, Ar-H), 7.85 (2H, dd, *J* = 7.8, 2.0 Hz, Ar-H), 7.51 (1H, dd, *J* = 8.8, 2.3 Hz, Ar-H), 7.46 (1H, dt, *J* = 9.2, 2.2 Hz, Ar-H); ^13^C NMR δ_C_ (101 MHz, DMSO-d_6_) 163.3 (dd, ^1^*J*_C-F_ = 247.0 Hz, ^3^*J*_C-F_ = 14.6 Hz), 148.6, 147.7, 138.8 (t, ^3^*J*_C-F_ = 13.2 Hz), 133.6, 131.9, 130.5, 129.3, 128.0, 124.9, 124.2, 122.9, 121.0, 119.8, 104.8, 104.5, 104.; Anal. calcd for: C_18_H_12_F_2_N_4_O_3_S, C, 53.73; H, 3.01; F, 9.44; N, 13.92; O, 11.93; S, 7.97. Found: C, 53.76; H, 3.06; F, 9.39; N, 13.87; O, 11.96; S, 7.96%. HRMS (m/z) [M + H]+ calcd 403.0671, found. 403.3818.

**6-(1-(2,3,4-Trifluorophenyl)-1*H*-1,2,3-triazol-4-yl)naphthalen-2-yl sulphamate 3L** Yield 26%; mp (with decomposition) 198-200 °C; ν_max_ (KBr)/cm^−1^ 3262, 3175, 3021, 1618, 1509, 1384, 915; ^1^H NMR δ_H_ (400 MHz, DMSO-d_6_) 9.27 (1H, d, *J* = 1.6 Hz, CH), 8.61 (1H, s, Ar-H), 8.20-8.07 (5H, m, Ar-H, NH_2_), 7.91-7.83 (2H, m, Ar-H), 7.67 (1H, qd, *J* = 9.8, 2.2 Hz, Ar-H), 7.50 (1H, dd, *J* = 8.9, 2.3 Hz, Ar-H); ^13^C NMR δ_C_ (101 MHz, DMSO-d_6_) 152.4–152.1 (m), 149.9-149.6 (m), 148.6, 147.3, 145.9-145.6 (m), 143.3-143.0 (m), 141.7-141.2 (m), 139.2–138.7 (m), 133.5, 131.9, 130.5, 129.2, 127.9, 125.0, 124.3, 123.8 (d, ^4^*J*_C-F_ = 3.3 Hz), 123.0 (dd, ^3^*J*_C-F_ = 8.3, ^4^*J*_C-F_ = 3.7 Hz), 122.9, 121.1 (dd, ^3^*J*_C-F_ = 8.5, ^4^*J*_C-F_ = 4.0 Hz), 119.7, 113.8 (dd, ^2^*J*_C-F_ = 18.6, ^3^*J*_C-F_ = 3.8 Hz); Anal. calcd for: C_18_H_11_F_3_N_4_O_3_S, C, 51.43; H, 2.64; F, 13.56; N, 13.33; O, 11.42; S, 7.63. Found: C, 51.39; H, 2.71; F, 13.51; N, 13.29; O, 11.48; S, 7.62%. HRMS (m/z) [M + H]^+^ calcd 421.0577, found. 421.3945.

### Molecular docking

#### Ligands and molecular target preparation

The 3 D structures of the potential steroid sulphatase inhibitors (ligands) were prepared using Portable HyperChem 8.0.7 Release (Hypercube, Inc., Gainesville, FL, USA)^29^. Before docking calculations, the structures of all ligands were optimised with an MM + force field^30^ and the Polak-Ribière conjugate gradient algorithm (terminating at a gradient of 0.05 kcal·mol^−1 ^Å^−1^). The X-ray structure of human STS was obtained from Protein Data Bank (accession code 1P49) and was prepared using standard procedure. Initially, the NAG, BOG, PO_4_^3–^ and water molecules from crystallisation were removed from the structure, and the catalytic amino acid fGly75 was converted to *gem*-diol form using the Maestro Protein Preparation Wizard module (Schrödinger, LLC, New York, NY, USA)^31^. Then, hydrogen atoms were introduced into the structure, and prepared model of the enzyme was optimised using the OPLS-AA force field^32^.

#### Molecular docking

Docking calculations of the optimised ligands to the prepared structure of human STS were carried out with AutoDock Vina 1.1.2 software (The Molecular Graphic Laboratory, The Scripps Research Institute, La Jolla, CA, USA)^33^. For all of docking studies, a grid box was centred on the Cβ atom of amino acid 75 of the prepared STS structure, and the size of the grid box was 24 Å × 24 Å × 24 Å. Then, the best binding modes for a particular ligand were inspected visually. Illustrations of the 3D models were prepared using VMD 1.9 (University of Illinois at Urbana-Champaign, Urbana, IL, USA)^34^. Identification of the ligand-protein interactions was performed using Discovery Studio Visualiser v20. 1. 0. 19295 (BIOVIA, Dassault Systémes, San Diego, CA, USA)^35^.

### Biological assays

#### *In vitro* STS assay

The STS enzyme was extracted from the human placenta, and purified using the 3-step chromatographic procedure according to the described method^36^. The reaction mixtures, at a final volume of 100 µL, containing 20 mM Tris-HCl pH = 7.4, 3 nM radiolabelled [^3^H] oestrone sulphate, various concentrations of inhibitor and 5 U of purified enzyme (1 U is the amount of enzyme that hydrolyses 100 nM radiolabelled [^3^H] oestrone sulphate in 1 h at 37 °C). The reactions were performed at 37 °C for 30 min. Afterwards, the reaction mixtures (90 µL) were collected from each well, and the product formed by STS-mediated hydrolysis of [^3^H]E1 was extracted with toluene (0.5 ml). Next 0.3 ml of toluene was combined with 0.3 ml of scintillation liquid. STS activity was measured using the radioluminometer MicroBeta (Perkin Elmer). Assays were performed in triplicate.

#### *In vitro* cellular assay using MCF-7 breast cancer cell line

Inhibition of STS activity in the MCF-7 breast cancer cell line was measured according to the method described by Purohit et al. ^37^. MCF-7 cells were maintained in Dulbecco’s Modified Eagle Medium supplemented with 10% of foetal bovine serum and cultured until 80% of confluence was received. Cells were seeded in 24-well microplates (Nest Biotechnology) at a density of 1·10^5^ cells/well. In order to assure an equal amount of cells in each reaction sample the number of cells was determined using a Bürker Counting Chamber. The STS activity was evaluated in living MCF-7 cells. Incubation of cells was conducted for 20 h at 37 °C in a 5% CO_2_ humidified incubator in a serum-free medium (0.5 ml) with radiolabelled oestrone sulphate [^3^H]E1S (4·105 cpm, 3 nM) with or without an inhibitor. After incubation, a medium containing STS-mediated reaction product was collected (0.45 ml) from each well and the product was isolated from the mixture by extraction with toluene (4 ml). The STS activity expressed as the level of product radioactivity was measured using the Radioluminometer MicroBeta (Perkin Elmer). Cellular assay was carried out in triplicate. IC_50_ values were calculated using GraphPad Prism software.

## Results and discussion

### Molecular docking

Initially, to verify that the 6–(1-phenyl-1*H*-1,2,3-triazol-4-yl)naphthalen-2-yl sulphamate derivatives are able to effectively bind to the STS active site, molecular docking studies were performed. The X-ray structure of STS was retrieved from the Protein Data Bank (Protein Data Bank accession code 1P49) and properly prepared for docking calculations. The docking procedure of the optimised ligands was performed using AutoDock Vina 1.1.2 software (Molecular Graphics Laboratory, The Scripps Research Institute, LaJolla, CA). The calculated results for the proposed structures of the inhibitors **3A-L** were at a satisfactory, comparable level in the range of −6.0 to −8.3 kcal·mol^−1^ (the measurement error for the AutoDock Vina software is 2.85 kcal·mol^−1^) (Table 1) and lower than the free energy of binding value of the reference inhibitor *Irosustat* (–5.4 kcal·mol^−1^). The most favourable binding energy was determined for compound **3L** (–8.3 kcal·mol^−1^) suggesting that this compound could theoretically create the most stable inhibitor-enzyme complex in the STS active site, leading to effective inhibition.

**Table 1.** Free energies of binding calculated for compounds **3 A-L** and *Irosustat*.


No.	R_1_	R_2_	R_3_	R_4_	R_5_	Free energies of binding [kcal·mol^-1^]
**3A**	H	H	H	H	H	–7.4
**3B**	H	F	H	H	H	–7.7
**3C**	H	Cl	H	H	H	–6.4
**3D**	H	Br	H	H	H	–6.3
**3E**	H	H	F	H	H	–7.6
**3F**	H	H	Cl	H	H	–7.6
**3G**	H	H	Br	H	H	–6.0
**3H**	F	H	H	H	H	–8.0
**3I**	Cl	H	H	H	H	–7.9
**3J**	Br	H	H	H	H	–8.0
**3K**	H	F	H	F	H	–7.9
**3L**	F	F	F	H	H	–8.3
*Irosustat*	–	–	–	–	–	–5.4

**Figure 1.**
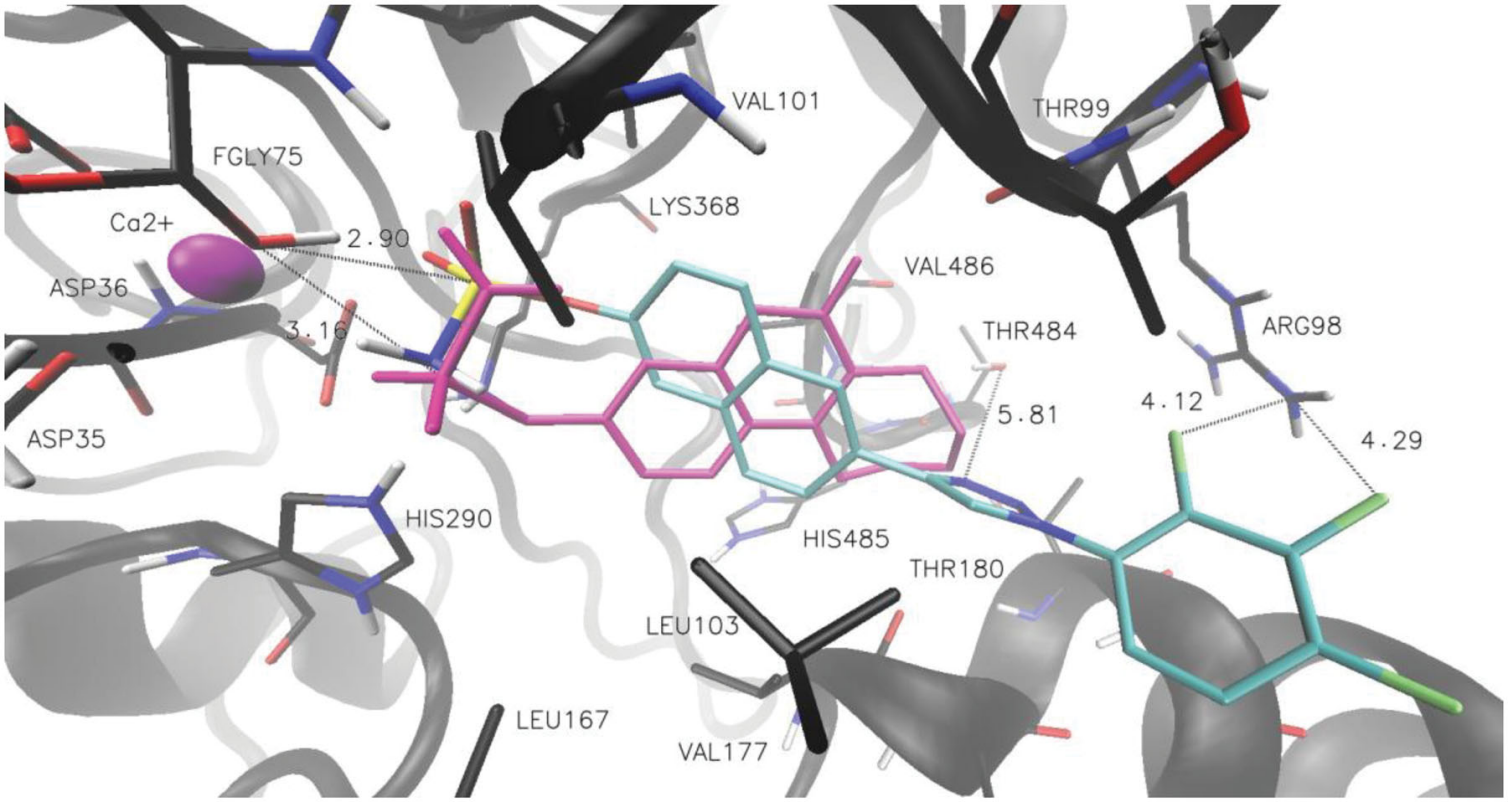
Docked binding modes and distance to fGly75, Arg98 and Thr484 for compound **3 L** (CPK coloured) and *Irosustat* (pink).

In order to analyse the ligand-protein interactions that could be responsible for the stabilisation of the inhibitor-enzyme complexes, studies using BIOVIA, Dassault Systémes, Discovery Studio Visualiser software have been carried out. Our research has shown that the newly designed potential STS inhibitors based on 6–(1-phenyl-1*H*-1,2,3-triazol-4-yl)naphthalen-2-yl sulphamate derivatives were able to create the complexes with the STS protein stabilised by a number of interactions including π-alkyl, alkyl, π-sulphur, conventional hydrogen bond, carbon hydrogen bond, π-cation, or π-sigma (listed in Table 2). The largest number of interactions was detected for compounds **3E**, **3F**, **3G**. However, apart from the above-mentioned numerous interactions, an extremely important aspect (influencing the inhibitory properties of STS inhibitors based on aryl-sulphamate derivatives) is their ability to undergo the nucleophilic substitution reactions on the sulphur atom. Although the mechanism of action is not confirmed and remains a topic of discussion, a sulphamate functional group (sulphate mimic) might be transferred to fGly75 residue leading to irreversible inhibition of the STS enzyme^21^. The visualisation of the putative binding mode for compounds **3L** using VMD 1.9 (University of Illinois at Urbana-Champaign, Urbana, IL, USA) is shown in Figure 1. The sulphamate functional group, which is directly responsible for the inactivation of the enzyme, is located in the catalytic region of STS close to the fGly75 residue coordinated to Ca^2+^ and stabilised by π-sulphur interaction with His290 (the distance between the sulphur atom of **3L** and OH group of fGly75 is 2.90 Å). For this reason, we suppose that the compound **3L** (despite a smaller number of electrostatic interactions indicated by the docking program) may prove to be the most effective due to the very close distance of the sulphamate group to fGly75 residue. Furthermore, the tetracyclic core of compound **3L** is well accommodated in the STS active site and is surrounded by some hydrophobic amino acid residues (e.g. Leu103, Leu167, Phe178, Phe182, Phe237, Val486, Phe488, and Phe553). Interestingly, the fluorine atoms of compound **3L** are within a short distance to the nitrogen atoms of the Arg98 residue (4.12 and 4.29 Å), indicating the possibility of electrostatic interactions (undetected by the Discovery Studio Visualiser). On the other hand, the presence of the fluorine atoms may be crucial for its potentially increased ability to undergo the enzymatic reaction. Highly electronegative fluorine atoms may reduce the pKa value of the molecule, making it a good leaving group in the nucleophilic substitution reaction on the sulphur atom. In addition, the molecular modelling studies indicated (Figure 1) that the triazole ring of compound **3L** is located close to the Thr484 residue, suggesting an additional interaction including hydrogen bond between OH group of Thr484 and ring-nitrogen atom (5.81 Å). These detected interaction points may be responsible for an enhancement of inhibitory potency by stabilisation of the potential STS inhibitor in the enzyme’s active site.

**Table 2.** The ligand-protein interactions (and distances [Å]) identified using BIOVIA, Dassault Systémes, Discovery Studio Visualiser.


No.	π–Alkyl	Alkyl	π–Sulphur	Conventional Hydrogen Bond	Carbon Hydrogen Bond	π–Cation	π–Sigma
**3A**	LEU103 (6.16, 6.50); VAL486 (5.86); VAL101 (4.93, 6.33); VAL177 (6.12)	–	HIS290 (5.76); HIS136 (6.93)	THR165 (4.11)	–	–	–
**3B**	LEU103 (6.13, 6.47); VAL486 (5.50, 6.07), VAL101 (5.31), VAL177 (5.50, 5.57)	–	HIS290 (6.00)	FGLY75 (4.18), LYS368 (5.02)	HIS136 (5.74, 7.12)	–	–
**3C**	LEU103 (5.91); VAL486 (6.16); VAL101 (5.43); VAL177 (5.43, 5.63)	ARG98 (2.99), TRP550 (6.97)	HIS290 (5.75); HIS136 (7.13)	–	HIS136 (6.04)	ARG98 (4.40)	–
**3D**	LEU103 (5.51); VAL101 (4.85); VAL177 (4.85, 5.55)	ARG98 (3.08); TRP550 (6.86)	HIS290 (5.79); HIS136 (7.11)	–	–	ARG98 (4.83)	
**3E**	ARG98 (4.57, 6.35); VAL177 (6.26); VAL486 (5.36), VAL101 (6.07)	–	HIS290 (5.43); HIS136 (6.78)	LYS134 (6.65); ASP36 (4.96); GLN343 (5.61)	THR165 (5.41)	–	VAL101 (4.93)
**3F**	ARG98 (4.74); VAL177 (6.49); VAL101 (5.81), VAL486 (5.37)	–	HIS290 (5.29); HIS136 (6.73)	LYS134 (5.37), THR165 (4.20)	–	–	VAL101 (4.78)
**3G**	ARG98 (4.95); VAL177 (5.24, 5.96), VAL486 (5.30), VAL101 (6.42)	LEU185 (5.88)	HIS290 (4.87, 5.32); HIS136 (6.72)	LYS368 (5.58), LYS134 (6.45), GLN343 (5.45), ASP36 (4.95), ASP35 (4.95)	HIS136 (5.58)	–	VAL101 (5.29)
**3H**	LEU103 (6.23, 6.49); VAL486 (6.21); VAL101 (5.38); VAL177 (5.49, 5.69)	–	HIS290 (5.96); HIS136 (7.09)	FGLY75 (4.25, 5.14); LYS368 (3.69)	HIS136 (5.74)	–	–
**3I**	PHE178 (4.53); LEU103 (6.27, 6.56); VAL177 (5.46, 5.79); VAL486 (5.96), VAL101 (5.42)	–	HIS290 (6.01); HIS136 (7.08)	LYS368 (5.15)	HIS136 (5.73)	–	–
**3J**	PHE178 (4.48); LEU103 (6.15, 6.43), VAL177 (5.50, 5.67); VAL486 (6.08); VAL101 (5.34)	–	HIS290 (5.92); HIS136 (7.05)	LYS368 (5.18); FGLY75 (3.04, 4.27)	HIS136 (5.69)	ARG98 (4.90)	–
**3K**	LEU103 (6.18, 6.60); VAL486 (5.95); VAL101 (5.40); VAL177 (5.26, 5.68)	–	HIS290 (5.91); HIS136 (7.05)	ARG98 (5.14)	HIS136 (5.67)	–	–
**3L**	VAL177 (4.32); VAL486 (5.09, 5.61); LEU74 (6.53)	–	HIS290 (5.57)	–	–	–	VAL101 (5.12)
*Irosustat*	VAL177 (6.51); VAL486 (4.87, 5.08) VAL101 (5.09)	–	HIS290 (5.73); HIS136 (6.84)	LYS368 (5.49); FGLY75 (3.04)	HIS136 (6.03)	–	VAL486 (4.67)

### Chemistry

**Scheme 1.**
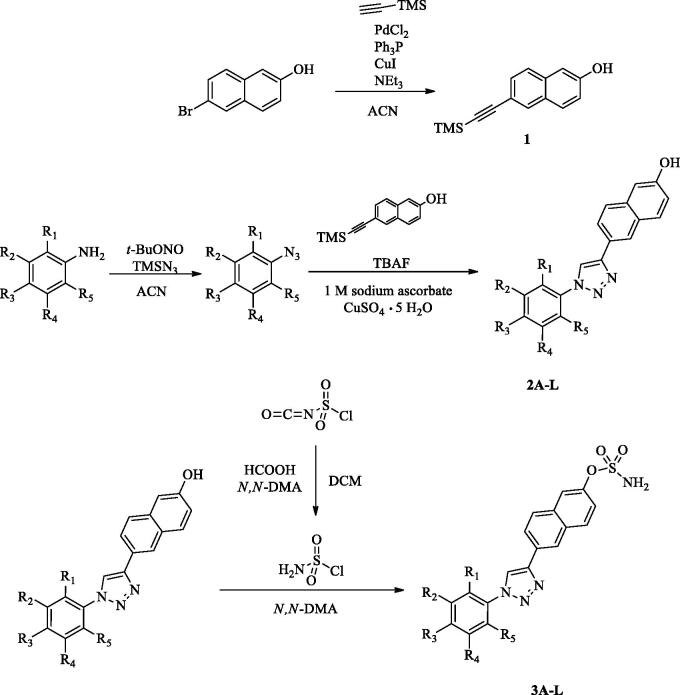
Synthesis of 6-((trimethylsilyl)ethynyl)naphthalen-2-ol **1** and 6–(1-phenyl-1*H*-1,2,3-triazol-4-yl)naphthalen-2-yl sulphamate derivatives (R_1_, R_2_, R_3_, R_4_, R_5_ = H, F, Cl, or Br) **3 A-L**.

The newly designed compounds **3A-L** based on 6–(1-phenyl-1*H*-1,2,3-triazol-4-yl)naphthalen-2-yl sulphamate derivatives were synthesised according to the route shown in (Scheme 1). In the first step, we synthesised 6-((trimethylsilyl)ethynyl)naphthalen-2-ol **1** by the Sonogashira coupling reaction between 6-bromo-2-naphthol and trimethylsilylacetylene. Next step included the preparation of 6–(1-phenyl-1*H*-1,2,3-triazol-4-yl)naphthalen-2-ol derivatives **2 A-L** by a 1,3-dipolar cycloaddition reaction. In this case the corresponding azide derivatives (obtained from commercially available anilines by the reaction with *t*-BuONO and TMSN_3_) were treated with **1** in the presence of CuSO_4_ · 5H_2_O (0.1 equiv.), sodium ascorbate (0.2 equiv.), and TBAF. Finally, 6–(1-phenyl-1*H*-1,2,3-triazol-4-yl)naphthalen-2-ol derivatives **2 A-L** were sulphamoylated using sulphamoyl chloride previously generated in the reaction of chlorosulphonyl isocyanate and formic acid in the presence of a catalytic amount of *N*,*N*-DMA. After standard isolation, the desired derivatives **3A-L** were obtained.

### Biological evaluation

#### Enzymatic assay using the STS enzyme isolated from the human placenta

In the next step of our investigation, the synthesised new STS inhibitors based on 6–(1-phenyl-1*H*-1,2,3-triazol-4-yl)naphthalen-2-yl sulphamate derivatives **3A-L** were tested in the enzymatic assay (at 0.5 µM of inhibitor concentration) in order to verify their theoretical potential to inhibit of the STS activity. Screening tests were performed using the STS enzyme isolated from the human placenta and purified by a 3-step chromatography procedure. After purification, the obtained fraction was used directly as the enzyme source. In this activity assay, a radiolabelled [^3^H] oestrone sulphate has been used as a substrate to provide higher selectivity and more reliable results. The summarised results of the enzymatic assay for newly synthesised 6–(1-phenyl-1*H*-1,2,3-triazol-4-yl)naphthalen-2-yl sulphamate derivatives **3A-L** are presented in Table 3. The received data showed that all compounds effectively inhibited the activity of the steroid sulphatase to a level from 7.98 to 27.17%. In the course of the research, we found that the highest inhibitory activity was exhibited by the compound **3L** containing three fluorine atoms in its structure (remaining STS activity of 7.98%). This result was in agreement with the data from the molecular modelling studies. It was observed that the substitution of the terminal aromatic ring with a less halogen atoms resulted in a decrease in the activity of the tested compounds towards STS. This observation may confirm our assumptions that the higher number of electronegative heteroatoms may reduce the pKa value of the inhibitor cores, making them more susceptible to nucleophilic substitution reaction on the sulphur atom. The obtained results may also suggest that the position of the halogen substitution in the above-mentioned aryl ring may be crucial for the potency of evaluated compounds. As it turns out, the presence of halogen atoms at the R_1_ and R_2_ positions may have the greatest influence on the increase of inhibitory potency. On the other hand, the type of substituent is slightly less significant.

**Table 3.** The STS inhibitory effect of the newly synthesised compounds **3 A-L** at 0.5 *µ*M inhibitor concentration.


No.	R_1_	R_2_	R_3_	R_4_	R_5_	Remaining STS activity [%]
**3A**	H	H	H	H	H	24.50 ± 1.36
**3B**	H	F	H	H	H	17.40 ± 0.87
**3C**	H	Cl	H	H	H	9.66 ± 0.48
**3D**	H	Br	H	H	H	9.62 ± 0.48
**3E**	H	H	F	H	H	23.75 ± 1.22
**3F**	H	H	Cl	H	H	18.71 ± 0.94
**3G**	H	H	Br	H	H	23.07 ± 1.15
**3H**	F	H	H	H	H	27.17 ± 1.36
**3I**	Cl	H	H	H	H	16.93 ± 0.83
**3J**	Br	H	H	H	H	15.93 ± 0.80
**3K**	H	F	H	F	H	14.03 ± 0.70
**3L**	F	F	F	H	H	7.98 ± 0.40

#### Evaluation of STS inhibition in the MCF-7 cell line

The next step of the biological evaluation was to verify whether the obtained new STS inhibitors based on 6–(1-phenyl-1*H*-1,2,3-triazol-4-yl)naphthalen-2-yl sulphamates **3A-L** are able to inhibit STS activity in the MCF-7 cancer cell line. In the course of our study, we determined the level of STS inhibition in the MCF-7 cells after incubation in the presence of inhibitors at 100, 10, and 1 nM concentrations. The summarised results are presented in Table 4. In the course of our research, we found that in the presence of inhibitor at 100 nM concentration, the lowest STS activities were measured for derivatives **3K** (5.43% of STS activity) and **3L** (5.29% of STS activity) substituted at R_1_, R_2_ or R_3_ position with fluorine atoms. These results were comparable with those obtained for the reference compound – *Irosustat* (remaining STS activity of 5.72%). In the next step, six of the most promising derivatives **3G-L** were selected and tested at an inhibitor concentration of 10 nM. Among these compounds, the highest STS inhibitory activities were observed for the derivatives **3I**, **3K**, and **3L** (remaining STS activity of 42.09%, 42.01% and 27.48%, respectively). As the research showed, the tested compounds showed slightly weaker activity in comparison with *Irosustat* (remaining STS activity of 12.93% at an inhibitor concentration of 10 nM). In order to further verify the potential of the new selected compounds, the STS activity was measured in the presence of **3I**, **3K**, and **3L** at the inhibitor concentration of 1 nM. For these compounds, an IC_50_ parameter was determined as well. The studies showed that the tested compounds based on 6–(1-phenyl-1*H*-1,2,3-triazol-4-yl)naphthalen-2-yl sulphamates **3I**, **3K**, and **3L** found to be very potent STS inhibitors characterised by low IC_50_ values of 30.14, 17.02, 15.97 nM, respectively (IC_50_ parameter determined for *Irosustat* was 1.14 nM). The obtained results indicated that 6–(1-phenyl-1*H*-1,2,3-triazol-4-yl)naphthalen-2-yl sulphamates may be very promising in further *in vivo* studies. Overall, among all the newly prepared compounds, the highest activity was shown by those with fluorine atoms in their structures. We assume that their effectiveness may be influenced by the possibility of electrostatic interactions between fluorine atoms and the Arg98 residue in the STS active site (as suggested in molecular modelling studies) as well as a higher susceptibility of these structures to nucleophilic substitution reaction on the sulphur atom. Moreover, the presence of C-F bonds in the structure of biologically active compounds often affects a number of favourable properties, including metabolic stability, leading to higher therapeutic effectiveness.

**Table 4.** Remaining STS activity in MCF-7 cells after incubation with compounds **3 A-L** and *Irosustat* at 100, 10 and 1 nM inhibitor concentrations.


No.	R_1_	R_2_	R_3_	R_4_	R_5_	Remaining STS activity [%]			
						100 [nM]	10 [nM]	1 [nM]	IC_50_ [nM]
**3A**	H	H	H	H	H	15.52 ± 0.78	–	–	–
**3B**	H	F	H	H	H	12.22 ± 0.61	–	–	–
**3C**	H	Cl	H	H	H	22.05 ± 1.10	–	–	–
**3D**	H	Br	H	H	H	20.59 ± 1.03	–	–	–
**3E**	H	H	F	H	H	11.32 ± 0.57	–	–	–
**3F**	H	H	Cl	H	H	13.25 ± 0.66	–	–	–
**3G**	H	H	Br	H	H	8.68 ± 0.43	59.86 ± 2.99	–	–
**3H**	F	H	H	H	H	8.80 ± 0.44	55.84 ± 2.79	–	–
**3I**	Cl	H	H	H	H	7.44 ± 0.37	42.09 ± 2.10	84.32 ± 4.22	30.14 ± 1.51
**3J**	Br	H	H	H	H	7.30 ± 0.37	59.45 ± 2.97	–	–
**3K**	H	F	H	F	H	5.43 ± 0.27	42.01 ± 2.10	79.30 ± 3.96	17.02 ± 0.85
**3L**	F	F	F	H	H	5.29 ± 0.26	27.48 ± 1.37	99.00 ± 4.95	**15.97 **±** 0.80**
*Irosustat*	–	–	–	–	–	5.72 ± 0.29	12.93 ± 0.65	16.83 ± 0.84	1.14 ± 0.06

## Conclusions

In the present work, we described our research on molecular modelling, synthesis, and biological evaluation of 6–(1-phenyl-1*H*-1,2,3-triazol-4-yl)naphthalen-2-yl sulphamate derivatives **3A-L** as new very potent STS inhibitors. Screening enzymatic assay, performed using the STS enzyme isolated from the human placenta indicated that all of the newly synthesised inhibitors **3A-L** were able to effectively inhibit the action of STS. Among them, the highest inhibitory activity was exhibited by compound **3L** containing three fluorine atoms in its structure (remaining STS activity of 7.98%). In the course of the cell line experiment, we observed the highest inhibition of STS in the presence of 6–(1-phenyl-1*H*-1,2,3-triazol-4-yl)naphthalen-2-yl sulphamates **3I**, **3K**, and **3L** characterised by low IC_50_ values of 30.14, 17.02, 15.97 nM, respectively (IC_50_ value determined for *Irosustat* was 1.14 nM). The presented results showed that 6–(1-phenyl-1*H*-1,2,3-triazol-4-yl)naphthalen-2-yl sulphamate derivatives may be very promising anticancer agents and their therapeutic potential should be confirmed in further *in vivo* studies. Furthermore, the data of enzymatic and cell line experiments suggested that the possibility of creating electrostatic interactions between the fluorine atoms of compounds and the Arg98 residue in the active site of STS as well as a higher susceptibility to nucleophilic substitution reaction on the sulphur atom could be critical for their inhibitory effects.
